# Co-infection of intestinal parasites and *Helicobacter pylori* among upper gastrointestinal symptomatic adult patients attending Mekanesalem Hospital, northeast Ethiopia

**DOI:** 10.1186/s13104-018-3246-4

**Published:** 2018-02-20

**Authors:** Abdurahaman Seid, Zemenu Tamir, Brhanu Kasanew, Moges Senbetay

**Affiliations:** Po. Box 1145, Dessie, Ethiopia

**Keywords:** Co-infection, *H. pylori*, *G. lamblia*, Ethiopia

## Abstract

**Objective:**

Intestinal parasites and *H. pylori* are well-known for their high prevalence worldwide. Thus, the objective of this study waste assess risk factors and co-infection of intestinal parasites and *H. pylori* among adult patients with upper gastrointestinal complaints. A hospital-based cross sectional study was conducted among 363 consecutive adult patients from December 10, 2015 to February 30,2016. Stool and venous blood were collected for analysis of Intestinal parasites and *H. pylori* infection, respectively. Data was analyzed using SPSS version 16 and logistic regression analysis was carried out to assess predictors of co-infection. A *p* ≤ 0.05 was considered as statistically significant.

**Results:**

*Helicobacter pylori* IgG and intestinal parasites were detected in 70.25–38.3% of participants, respectively while *G. lamblia* accounted 22.3%*. G. lamblia* prevalence was significantly higher among *H. pylori* infected participants (COR: 2.76; 95% CI: 1.46–5.23), but *E. hystolytica/dispar* infection didn’t show significant variation (p = 0.15). *H. pylori* and intestinal parasites concomitant co-infection was associated with male sex (AOR: 1.61; 95% CI: 1.01–2.56), consumption of river water (AOR: 1.85; 95% CI: 1.11–3.07) and ground/spring water (AOR: 4.10; 95% CI: 1.97–8.52). Thus, besides *H. pylori* investigation, upper gastrointestinal symptomatic patients should be screened for *G. lamblia* infection and other intestinal parasites.

## Introduction

Gastrointestinal symptoms are highly prevalent in the general population. Dyspepsia is the most common upper gastrointestinal symptom complex [1–3] with 25% of the general population suffering from dyspeptic symptoms [4, 5]. The causes of dyspepsia remain uncertain and are likely to be infectious and non-infectious agents. *Helicobacter pylori* (*H. pylori*)*, Giardia lamblia (G. lamblia*)]and coeliac disease are common causes of dyspepsia [6]. Recently, polymicrobial causes of upper gastrointestinal disorders have gained tremendous clinical significance, and the importance of synergism has been identified as significant contributor of dyspepsia [7].

It is estimated that *H. pylori* infects more than 50% of the world population [8] with highest burden among developing countries like those in Africa [9]. *G. lamblia* has also a worldwide distribution affecting approximately 200 million people globally with 500,000 new cases per year [10]. Besides similar mode of transmission and strong co-relation to socioeconomic levels [11], urease production by *H. pylori* helps intestinal parasites and bacteria to easily cross the stomach’s acid environment [12].

Besides *H. pylori* infection, *G. lamblia* has been found to trigger symptoms of gastrointestinal disorders [13, 14] with dyspepsia reported to be the main symptoms of gastric giardiasis [15]. Similarly, 30% and 15.8–44% of *H. pylori* and *G. lamblia* infection, respectively was reported among patients with dyspepsia [6, 11]. Children with gastric complaints showed 65, 10–25% of *H. pylori* infection, giardiasis and amoebiasis, respectively [16]. In different parts of the world, a potential association between intestinal protozoans and *H. pylori* infection was documented [6, 17, 18] with 75% prevalence of *H. pylori* infection among patients with gastric giardiasis [16]. This suggests that health care professionals should be aware of gastric giardiasis as a possible cause of dyspepsia.

In Ethiopia, many studies reported high prevalence of *H. pylori* infection among dyspeptic patients [19–23], but information about rate of intestinal parasites infection is limited. Besides *H. pylori* infection, it is necessary to assess intestinal parasite infection status of dyspeptic patients to improve their clinical outcome. Therefore, the aim of this study was to assess concomitant co-infection of intestinal parasites and *H. pylori*, and associated risk factors of adult upper gastrointestinal symptomatic patients at Mekanesalem Hospital.

## Main text

### Methods

#### Study patients and setting

This cross-sectional study was conducted at Mekanasalem Hospital from December 10, 2015 to February 30, 2016. It is located in South Wollo zone, Amhara region, northeast Ethiopia. It is the only Hospital in the study area, with 200 beds and different units of both inpatient and outpatients services to the community. The minimum sample size was determined to be 384 by taking 5% marginal error, 95% CI, and 50% prevalence since there was no previous study on the current issue. The study population consisted of adult consecutive outpatients with complaints of one or more upper gastrointestinal symptoms with clinical indications of *H. pylori* infection.

#### Inclusion and exclusion criteria

Participants who had at least one of the following upper gastrointestinal complaints such as upper abdominal pain, abdominal bloating, nausea, vomiting, early satiation, post prandial fullness, heartburn, regurgitation, epigastric pain/burning, belching and being volunteer to participate were included. However, patients who were on antibiotic and anti-parasite drugs for 1 week prior to the study involvement, and those with mental problems and patients below 18 years old were excluded.

#### Data collection

Each study participants were asked to respond to a questionnaire containing sex, age, residence, marital status, education, occupation, and environmental conditions such as water supply and availability of toilet. About 5 ml of venous blood was collected from each participant and serum was separated for serological testing. Thereafter, each participant was given a pre-labeled clean and dry plastic container to collect fresh stool specimen so as to check for intestinal parasites.

#### Clinical specimen analysis

All collected stool samples were screened for intestinal parasites by using direct wet mount using 0.85% sodium chloride solution as recommended by standard guideline [24]. The blood samples were examined serologically for *H. pylori* immunoglobulin G (IgG) antibodies using immune chromatographic rapid test kits (dBest *H. pylori* test kit, Zhejiang, China), which is nationally approved and used for serological diagnosis of *H. pylori* infection. The manufacturers’ instruction was strictly followed for diagnosis of *H. pylori* infection.

#### Statistical analysis

Statistical analysis was performed with SPSS version 16.0. For the descriptive data, frequencies and percentages were used to describe the characteristics of the participants. To assess the association between dependent and independent variables, logistic regression analysis was used. Odds ratios (OR) and 95% confidence intervals (CI) were used to indicate association of the variables. A *p* ≤ 0.05 was considered statistically significant.

#### Ethics approval and consent to participate

Research Ethical approval was obtained from the research ethics committee of Wollo University, college of medicine and health sciences, and permission letters was obtained from the hospital management committee. Before commencement of data collection the purpose of the study was explained to the participants and all of them provided written informed consent. Intestinal parasite and *H. pylori* screening was performed free of charge, and those found to have infection were managed by physicians.

### Results

#### Socio-demographic characteristics

During the study period a total of 363 (57.3% females) with upper gastrointestinal symptomatic adults who fulfilled the inclusion criteria were included in the analysis. The age of participants ranged from 18 to 85 years with a mean (± SD) of 39.11 ± 15.38 years. Majorities, 38.3%, of the participants do not have formal education and 42.1% were farmer. Two hundred eight (57.3%) of total participants were rural residents, and 61.4% of participants had used tap water for their daily consumption. The socio-demographic characteristic of study participants is summarized in Table 1.

**Table 1:** Socio-demographic characteristics of study participants attending at Mekanesalem Hospital from December 10, 2015 to February30, 2016 (N = 363)

Variable	Frequency	%
Sex		
Male	155	42.7
Female	208	57.3
Age in years		
≤ 20	36	9.9
21–30	103	28.4
31–40	84	23.1
41–50	63	17.4
> 50	77	21.2
Residence		
Urban	155	42.7
Rural	208	57.3
Marital status		
With partner	234	64.5
Without partner	129	35.5
Education		
Illiterate	139	38.3
Primary school (1–8 grade)	72	19.8
Secondary school (9–12 grade)	64	17.6
College and above	88	24.2
Occupation		
Farmer	153	42.1
Student	71	19.6
House wife	40	11
Government employee	68	18.7
Others	31	8.5
Drinking water		
Tap water	223	61.4
River	104	28.7
Ground/spring	36	9.9
Availability of toilet		
No	68	18.7
Yes	295	81.3

#### *Pylori* and intestinal parasites co-infection

Among 363 participants, *H. pylori* IgG and intestinal parasite (IP) were detected in 225 (70.25%) and 139 (38.3%) participants, respectively. *G. lamblia* accounted 22.3% of isolated parasites. Higher proportion of IP (44.3%) was detected among *H. pylori* infected participants. Univariate analysis showed statistically significant association between *G. lamblia* and *H. pylori* infection (COR = 2.76; 95% CI: 1.46–5.23). However, no significant association observed between *E. hystolytica*/diaper and *H. pylori* infection as shown in Table 2.

**Table 2:** Prevalence of *H. pylori* and intestinal parasites among study participants attending at Mekanesalem Hospital from December 10, 2015–February 30, 2016

	Total (%)	*H. pylori* infection		COR (95% CI)	p value
		No, no (%)	Yes, no (%)		
Intestinal parasite					
No parasite	224 (61.7)	82 (75.9)	142 (55.7)	1	
*G. lamblia*	81 (22.3)	14 (13)	67 (26.3)	2.76 (1.46–5.23)	0.002*
*E. hystolytica*	47 (12.9)	12 (11.1)	35 (13.7)	1.68 (0.83–3.43)	0.15
*A. lumbricoids*	4 (1.1)	–	4 (1.6)		
*E. vermicularis*	7 (1.9)	–	7 (2.7)		
Total	363 (100)	108 (100)	255 (100)		

* Significant

Univariate analysis showed statistically significant association between gender, residence, type of occupation, educational level, availability of toilet in the home/village, type of water source for daily consumption and *H. pylori*-IP co-infection. However, clearing the possible confounding factors, a multiple logistic regression model showed statistical significant association between male gender (AOR: 1.61; 95% CI: 1.01–2.56), river water consumption (AOR: 1.85; 95% CI: 1.11–3.07), ground/spring water consumption (AOR:4.10; 95% CI: 1.97–8.52) and *H. pylori*-IP co-infection (Table 3).

**Table 3:** Regression analysis showing factors associated with *H. pylori*-intestinal parasites co-infection

Variables	n	Co-infection	COR (95% CI)	AOR (95% CI)	p value
		Yes (%)			
Sex					
Female	208	55 (26.4)	1	1	
Male	155	57 (36.8)	1.62 (1.03–2.54)	1.61 (1.01–2.56)	0.044*
Residence					
Urban	155	33 (21.3)	1		
Rural	208	79 (38.0)	2.26 (1.41–3.64)	–	
Marital status					
Without partner	129	45 (34.9)	1	–	
With partner	234	67 (28.6)	0.75 (0.47–1.19)		
Gov’t employee	68	14 (20.6)	1		
Occupation					
Farmer	153	58 (37.9)	2.36 (1.20–4.61)	–	
Student	71	26 (36.6)	2.23 (1.04–4.77)	–	
House wife	40	10 (25.0)	1.29 (0.51–3.25)	–	
Other	31	4 (12.9)	0.57 (0.17–1.90)	–	
Education					
Illiterate	139	51 (36.7)	2.10 (1.14–3.89)	–	
Primary (1–8)	72	17 (23.6)	1.12 (0.53–2.36)	–	
Secondary (9–12)	64	25 (39.1)	2.33 (1.14–4.76)	–	
College and above	88	19 (21.6)	1		
Availability of toilet					
Yes	295	78 (26.4)	1		
No	68	34 (50)	2.78 (1.62–4.78)	–	
Drinking water					
Tap	223	53 (23.8)	1	1	
River	104	39 (37.5)	1.93 (1.17–3.18)	1.85 (1.11–3.07)	0.018*
Ground/spring	36	20 (55.6)	4.01 (1.94–8.29)	4.10 (1.97–8.52)	0.000*

* Significant

### Discussion

*Giardia lamblia*, *E. hystolytica/dispar* and *H. pylori* are considered the most common infectious agents affecting human beings in developing countries [25]. In our study, intestinal parasites were detected in 38.3% of upper gastrointestinal symptomatic patients with *G. lamblia* (22.3%) being the most prevalent. A similar study conducted in Mexico showed higher (48.4%) prevalence of IP infection with *E. histolytica/dispar* being the dominant (21.5%) parasite [26]. However, our result is comparable to a study conducted in Sudan where 22% of *G. lamblia* was detected among patients with gastrointestinal disorder [27].

In this study, high prevalence of IP (44.3%) was observed among *H. pylori* infected individuals compared to uninfected participants, which is in agreement to a recent report from Ethiopia where IP was significantly associated with *H. pylori* infection [20]. This is due to urease production by *H. pylori* that converts urea of the stomach wall to ammonia resulting in increment in stomach pH [28, 29] and allowing intestinal parasites to cross easily and reach to intestine. The possible similar routes of transmission such as the fecal–oral route could also explain the observed high prevalence of IP among *H. pylori* infected participants in our study.

Even though higher proportion of *E. histolytica/dispar* was observed among *H. pylori* infected than uninfected participants, this is not statistically significant. Similar to our result, Moreira et al. showed no significant association between *E. histolytica* infection and *H. pylori* sero-positivity [30]. However, Torres et al. showed significantly lower prevalence of *H. pylori* infection among adults carrying *E. histolytica* than without *E. histolytica* [26]. However, this needs well designed cohort type research to ascertain the association.

In our study, we found a significantly higher proportion of giardiasis in participants that also had *H. pylori* infection as shown in Table 2. Our result is comparable to other studies conducted elsewhere in the world with strong correlation of concomitant *G. intestinalis* and *H. pylori* infections [30–33]. Recently, Zylberberg *et al*. in US found that individuals colonized with *H. pylori* are independently associated with giardiasis [34]. A case control study showed no significant difference in *H. pylori* and other IP infection, but the combination of *H. pylori* infection and giardiasis was significantly higher among symptomatic patients compared to controls [18].

However, our result is in contrary to study conducted in Sudan with no statistically significant difference of *G. lamblia*–*H. pylori* co-infection among *H. pylori* infected and uninfected participants [27]. This could be due to less sample size of the Sudan study. Moreover, we investigate *H. pylori* infection using IgG antibody which may not able to detect recent infection. In our study, the large proportion of *H. pylori* and giardia co-infections could be due to the role of urease produced by *H. pylori.* It is found a significant increase in urease activity among coinfected (giardiasis and *H. pylori*) compared to mono (*G. lamblia*) infected individuals [35]. But it is still vague whether giardiasis increases the susceptibility to *H. pylori* or not, which warrants further case control cohort and molecular based study.

Our result showed statistical significant association between male gender, river as well as ground/spring water consumption and *H. pylori*-intestinal parasites co-infection. This agrees with study in Egypt as gender is significantly associated with *Giardia* and *H. pylori* co-infection [31]. Furthermore, community based study is needed in this area to identify and clarify the possible risk factors of co-infection.

#### Conclusion

The present study revealed that 38.3–70.2% of IP and *H. pylori* infection, respectively, with a strong correlation of concomitant *G. lamblia* and *H. pylori* infections. Gender and type of drinking water source was significantly associated with intestinal parasites and *H. pylori* concomitant co-infection. The observed association requires further study to investigate the observed relationship in more detail, and explanations of this association are required. Besides *H. pylori* investigation, screening of *G. lamblia* infection is recommended for successful management of upper gastrointestinal symptomatic patients.

#### Limitations

The results of this study cannot be representative of the general population due to the following limitations:

- The cross-sectional nature of the study has limitation to show real correlation of IP and *H. pylori* infection.
- *H. pylori* IgG detection may overestimate the prevalence of *H. pylori* infection.
- Direct wet mount preparation may under estimate the prevalence of IP.
- Prevalence of *H. pylori* and intestinal parasites coinfection could not be representative of the general population since only upper gastrointestinal symptomatic patients were included in the study.

## Abbreviations

AOR: adjusted odds ratio
COR: crude odds ratio
IgG: immunoglobulin G
IP: intestinal parasite
OR: odds ratio
